# The role of neuroimmunology subspecialty in disease management of patients with neuromyelitis optica spectrum disorder

**DOI:** 10.1186/s13023-026-04443-x

**Published:** 2026-06-12

**Authors:** Binbin Xue, Jia Li, Dewei Xie, Xu Zhang, Junhui Xia, Xiang Li, Jie Lin

**Affiliations:** 1 Department of Anesthesiology, The First Affiliated Hospital of Wenzhou Medical University, Wenzhou, Zhejiang China; 2 Department of Neurology, The First Affiliated Hospital of Wenzhou Medical University, Wenzhou, Zhejiang China

**Keywords:** Neuromyelitis optica spectrum disorder, AQP4-antibody, Neuroimmunology subspecialty, Monoclonal antibody, Annualized relapse rate, EDSS

## Abstract

**Background:**

Neuromyelitis optica spectrum disorder (NMOSD) is a severe autoimmune inflammatory disease of the central nervous system (CNS) that requires specialized management. Although the role of subspecialty management has been established in various neurological conditions, the impact of neuroimmunology subspecialty (NIS) management on NMOSD patient outcomes remains inadequately explored.

**Methods:**

A retrospective analysis was conducted on 188 NMOSD patients, stratifying them into groups based on their management pathway: initial visit in NIS, initial visit in non-neuroimmunology subspecialty (NNIS), NNIS-to-NIS transfer, and last visit in either NIS or NNIS. Primary outcome measures included annualized relapse rate (ARR), Expanded Disability Status Scale (EDSS) scores.

**Results:**

Patients initially presenting to NIS IV demonstrated a lower ARR (median: 0.39, IQR: 0.31–0.51, 95% confidence interval (CI): 0.33–0.45) compared to other groups at last follow-up. For transfer patients, ARR significantly decreased post-transfer (*p* < 0.001). Patients in NIS IV group had the lowest EDSS scores (median: 1.3, IQR: 1.0–2.0, 95% CI: 1.0–2.0) compared to other groups. Both initial presentation to NIS (OR:0.50, 95%CI:0.26–0.97, *p* = 0.039) and subsequent transfer to NIS management (OR:0.41, 95%CI:0.21–0.81, *p* = 0.010) were associated with lower EDSS scores.

**Conclusion:**

NIS management significantly improves outcomes in NMOSD patients, as evidenced by lower relapse rates, reduced disability scores, and higher utilization of advanced therapeutics.

**Supplementary Information:**

The online version contains supplementary material available at 10.1186/s13023-026-04443-x.

## Introduction

NMOSD is a severe inflammatory dymyelinating disorder affecting the CNS [1]. The identification of anti-Aquaporin-4 antibodies as pathogenic markers represented a crucial breakthrough in understanding NMOSD pathophysiology [2]. The disease manifests through six core syndromes: transverse myelitis (TM), optic neuritis (ON), area postrema syndrome (APS), cerebral syndrome, diencephalic syndrome, and brainstem syndrome [3]. Disease progression is often severe, with 20–33% of patients developing significant visual and motor disabilities following recurrent attacks. More concerning, approximately 23% of patients become wheelchair-dependent, and 9% succumb to the disease within five years of onset [4, 5].

The clinical presentation of NMOSD is highly heterogeneous. In 85% of adult patients, optic neuritis or myelitis is the sentinel attack [6]. APS, characterized by intractable nausea, vomiting and hiccups, affects roughly 20% of patients [7, 8]. This diversity in initial symptoms often leads patients to seek management across various medical specialties: those with visual symptoms typically present to Ophthalmology, while those with intractable hiccups frequently consult Gastroenterology. Additionaly, patients presenting with non-specific symptoms, such as cutaneous pruritus and pain, pose significant diagnostic challenges. This dispersion of initial management frequently result in delayed diagnosis and treatment initiation.

As neurological subspecialties—including cerebrovascular disease, neuroimmunology, and neurodegeneration—have become increasingly specialized, the NIS has emerged as a key component in the long-term management of NMOSD. Neuroimmunology subspecialists possess in-depth expertise in neuroimmune disorders, including NMOSD—spanning disease mechanisms, cutting-edge clinical diagnosis and management (e.g., early use of high-efficacy therapies), longitudinal patient monitoring (ARR, EDSS, MRI), and coordination of multidisciplinary support. By contrast, non-neuroimmunology clinicians may be less engaged with advances in this field, which can contribute to delays in diagnosis and treatment. However, current NMOSD management guidelines do not adequately emphasize the role of subspecialty care in optimizing patient outcomes.

While numerous studies have investigated prognostic factors in NMOSD patients, such as early treatment initiation and the application of novel immunosuppressive therapies [9–12], limited research had focused on the role of neuroimmunology subspecialists in disease treatment and long-term management. The study aimed to evaluate the impact of neuroimmunology subspecialists on the long-term prognosis of NMOSD patients.

## Methods

### Participants

This retrospective study collected demographic and clinical data from the First Affiliated Hospital of Wenzhou Medical University. The inclusion criteria were as follows: (1) fulfillment of the 2015 International Panel for NMOSD diagnosis (IPND) criteria, (2) follow-up for at least 6 months. The exclusion criteria were: (1) incomplete medical records, and (2) lost to follow-up.

NMOSD was diagnosed per the 2015 NMOSD IPND criteria [3]. For AQP4-IgG–positive cases, at least one core clinical characteristic was required. For AQP4-IgG–negative/unknown cases, diagnosis required ≥ 2 core clinical characteristics with MRI support and exclusion of alternative diagnoses. Diagnosis followed the 2015 IPND criteria, including: core clinical characteristics (optic neuritis, acute myelitis, area postrema syndrome, acute brainstem syndrome, symptomatic narcolepsy/diencephalic syndrome, and cerebral syndrome with NMOSD-typical MRI), AQP4-IgG serostatus, supportive MRI features, and exclusion of alternative diagnoses.

All NIS cases used for this study are only from the First Affiliated Hospital of Wenzhou Medical University.

### Procedure

The data collected included sex, age of onset, demyelinating clinical presentation at onset, total numbers of attacks, ARR at the last follow-up, EDSS at last the follow-up, immunosuppressive therapy (IST) information, whether the initial diagnosis is made in the neuroimmunology subspecialtists and whether there is a subsequent referral to the neuroimmunology subspecialtists. Previous studies have indicated that patients with NMOSD has a higher relapse rate during its early stages. Neuroimmunology subspecialists were defined as clinicians who had received systematic training in neuroimmune disorders and had at least 10 years of clinical experience in diagnosing and treating these conditions.

To clarify the patient grouping in our study, we defined categories based on the site of initial visit and subsequent follow-up trajectory. Patients were first classified by their initial visit (IV) location: NIS IV included those who began visit at the NIS clinic and continued follow-up there. Patients in the NIS IV group received their initial evaluation at the NIS, where the diagnosis was established and subsequent treatment was initiated. Whereas NNIS IV comprised patients whose first visit occurred at non-NIS clinics. Notably, a subset of NNIS IV patients later transferred their ongoing management to NIS during the study period, forming the Transfer group. For outcome analyses, we defined two final-visit (LV) cohorts based on the ultimate care destination: NIS LV encompassed all patients whose final documented follow-up occurred at NIS (including both NIS IV patients and Transfer patients), whereas NNIS LV comprised patients who maintained their follow-up exclusively at NNIS throughout the study period without transferring to NIS.

The location of initial attack was categorized into ON, TM, APS, other, multifocal lesions.

A new or worsening neurological symptoms lasting for more than 24 h, accompanied by new or worsening MRI lesions was detected which was recognized as a relapse. ARR was calculated as the number of relapses per year. If the onset time to IST initiation was less than six months, the pre-ARR was defined over a 6-month period. The EDSS was evaluated through the medical records by two different certified neurologists. An EDSS score of 5 or greater at the last follow-up was considered indicative of an unfavorable prognosis.

### Statistical analysis

Statistical analysis was performed using the Python (version: 3.11.6, packaged by Conda-Forge). Categorical data are presented as percentages and frequencies. Continuous data are presented as the mean and standard deviation (SD), and ranked data by the median and interquartile range (IQR). The Mann–Whitney U test or Student’s t-test for quantitative data, χ² test or Fisher’s exact test for qualitative data based on the normality. Missing data was replaced by the median or mean.

An ordinal logistic regression model was used to identify variables associated with EDSS at the last follow-up. Covariables including sex, onset age, total attacks, seropositive of AQP4-ab, location of initial attack, monoclonal antibody administration, initial visit in NIS, last visit in NIS, disease duration, and NNIS transfer were included in the logistic regression model. Univariate analysis was performed first, and variables with *p* < 0.05 were entered into the multivariable model.

Sensitivity analysis were performed as follows: (1) subsamples of female patients to exclude the sex bias, (2) subsamples of patients with AQP-ab seropositive to exclude the possible AQP4-ab bias, (3) subsamples of patients whose onset age less than 50 years to exclude the possible age bias.

To balance baseline characteristics, we applied propensity score overlap weighting (PSOW) using sex, age of onset, AQP4-ab status, utilization of monoclonal antibody, and disease duration as covariates. For each patient, the overlap weight was calculated as OW = PS × (1 − PS). Covariate balance before and after weighting was evaluated using absolute standardized mean differences (SMD), with SMD < 0.1 indicating adequate balance.

Statistical significance was set at two-tailed *p* < 0.05. Odds ratio (OR), hazard ratio (HR), and the associated 95% CI values were calculated.

## Results

### Characteristics of the study cohort

In our study, 188 patients were enrolled, of whom 159 were female (84.6%). The median age at onset was 42.0 years (range of 14 to 88 years), and the median disease duration was 66 months (range from 6 to 272 months). Initially, 46 patients visited the NIS directly, while 95 patients were transferred to NIS after initially visiting NNIS. At the last follow-up, 141 patients were in the NIS LV group, and 47 remained in the NNIS LV group. The transfer time varied, ranging from 1 to 195 months. Among all patients, 167 were seropositive for AQP4-ab, 12 were seronegative, and the serological status was unknown for the remaining 9 patients. The initial attack locations were distributed as follows: ON (*n* = 70), myelitis (*n* = 57), APS (*n* = 25), others (*n* = 13), and multifocal involvement (*n* = 23). A total of 60 out of 188 NMOSD patients received monoclonal antibody therapy. In the NIS IV group, 21/46 (45.7%) patients received monoclonal antibody therapy, while 39/142 (32.4%) patients in the NNIS IV group received such treatment. Among them, 33/95 (34.7%) patients transitioned from NNIS IV to NIS IV and subsequently received monoclonal antibody therapy. The median ARR and EDSS at the last follow-up were 0.48 (IQR 0.32–0.65) and 2.0 (IQR 1.0-3.5), respectively (Table 1).

**Table 1:** Demographic characteristic

Characteristics	Total	NIS IV	NNIS IV	*P* value
Sex	188	46	142	
Female, n(%)	159(84.6%)	43 (93.5%)	116 (81.7%)	0.091
Male, n(%)	29 (15.4%)	3 (6.5%)	26 (18.3%)	
Onset age, median (IQR), years	42.0 (29.0−54.3)	39.5 (28.5–49.5)	44.0 (29.3–55.0)	0.306
Disease duration, median, (IQR) months	66.0 (34.5–126.0)	52.0 (32.5−106.3)	73.5 (35.0−130.3)	0.198
Serology	179 (9 unknown)	43 (3 unknown)	136 (6 unknown)	
AQP4-ab seropositivity, n (%)	167 (93.3%)	43 (100%)	124 (91.2%)	0.220
AQP4-ab seronegativity, n (%)	12 (6.7%)	0	12 (8.8%)	
Location of initial attack				
ON, n	70	24	46	0.196
TM, n	57	10	47	
APS, n	25	5	20	
Others, n	13	3	10	
Multifocal location, n	23	4	19	
Monoclonal antibody, n (%)	60 (31.9%)	21 (45.7%)	39 (27.5%)	0.034
RTX	49	16	33	
RTX switch to INE	5	1	4	
RTX switch to SAT	2	1	1	
RTX switch to ORF	1	0	1	
INE	11	5	6	
Conventional IST (any exposure)				
Azathioproine	33	4	29	
Cyclophosphamide	2	0	2	
Chronic glucocorticoids	35	5	30	
Chronic IVIg	1	0	1	
Leflunomide	1	0	1	
MMF	73	19	54	
Tacrolimus	5	0	5	
ARR at last follow-up, median (IQR)	0.48 (0.32–0.65)	0.39 (0.31–0.51)	0.53 (0.34–0.71)	< 0.001
EDSS at last follow-up, median (IQR)	2.0 (1.0−3.5)	1.3 (1.0–2.0)	3.0 (2.0–4.0)	0.003
Favorable prognosis (EDSS less than 5)	157 (83.5%)	45 (97.8%)	112 (78.9%)	0.005

APS: area postrema syndrome; ARR: annuallized relapse rate; EDSS: Expanded Disability Status Scale; IQR: inter-quartile range; IST: immunosuppressive therapy; IVIg: intravenous immunoglobulin; MMF: Mycophenolate Mofetil; NIS IV: Initial visit in neuroimmunology subspecialty; NNIS IV: Initial visit in non-neuroimmunology subspecialty; ON: optic neuritis; TM: transverse myelitis;

P value: comparison between NIS IV and NNIS IV

### Impact of NIS management on relapse rates and disability outcomes

**Fig. 1:**
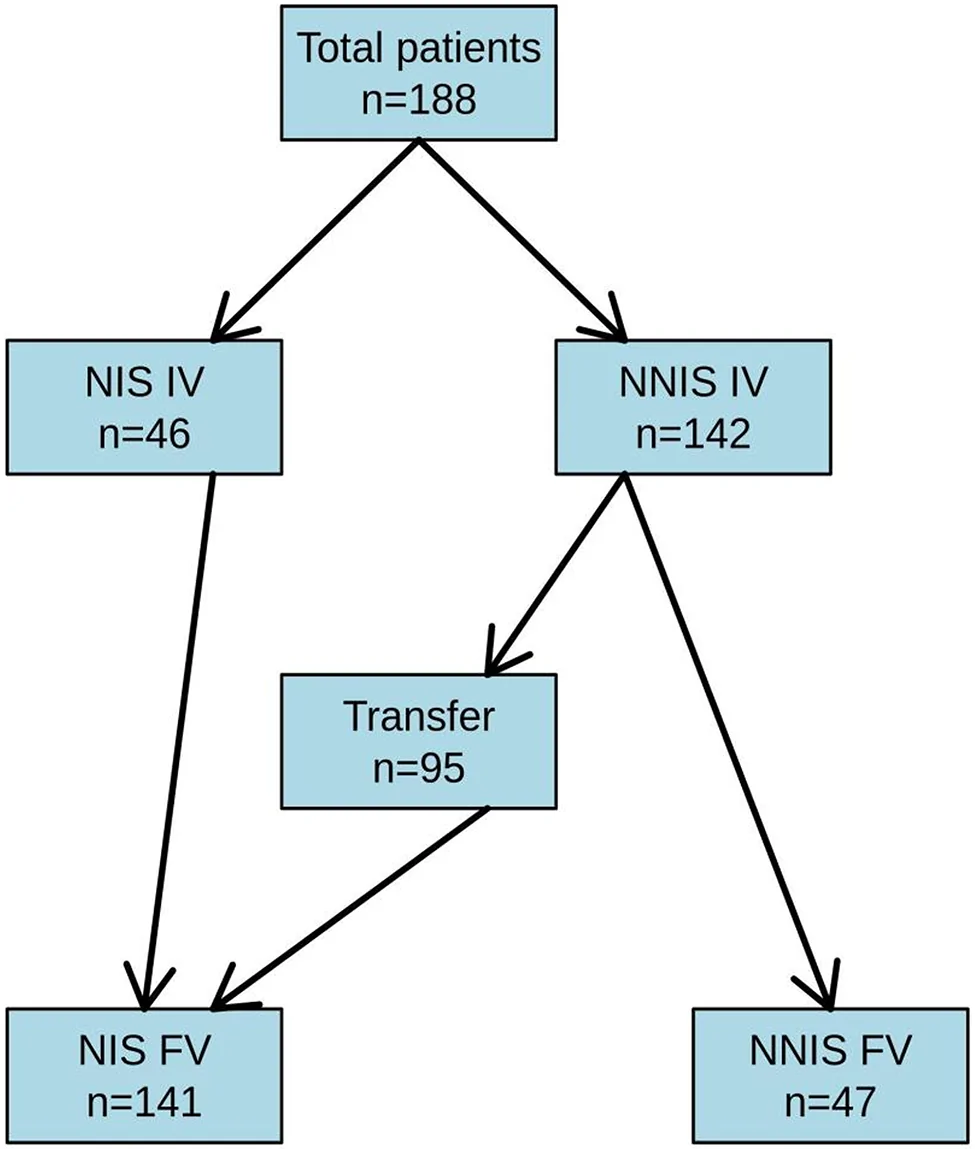
Flow chart. NIS IV: Initial visit in neuroimmunology subspecialty; NIS LV: Last visit in neuroimmunology subspecialty; NNIS IV: Initial visit in non-neuroimmunology subspecialty; NNIS LV: Last visit in non-neuroimmunology subspecialty

**Fig. 2:**
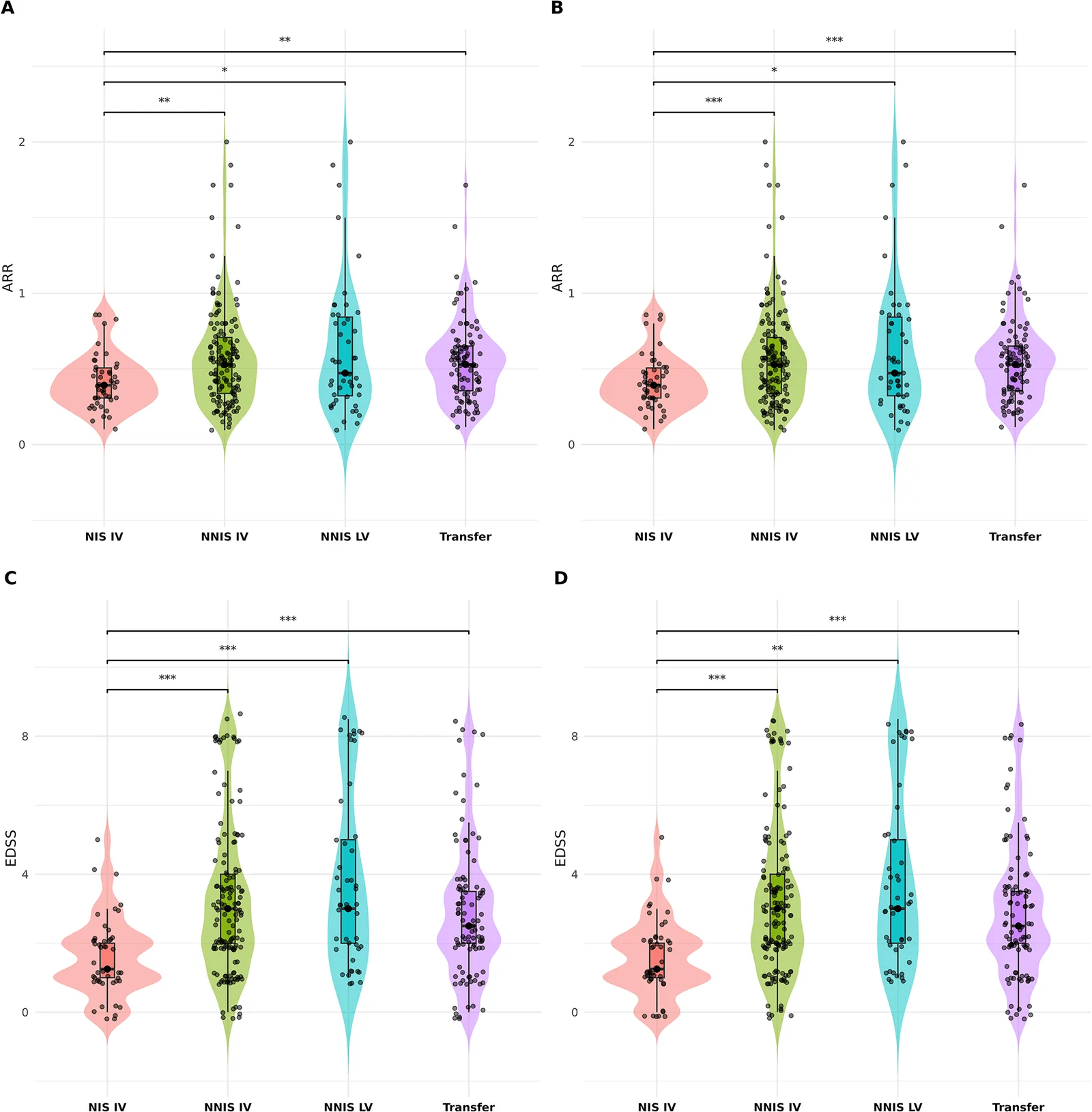
**A**: Comparison of ARR at the last follow-up across the four groups. **B**: Comparison of ARR at the last follow-up across the four groups after overlap weighting. **C**: Comparison of EDSS at the last follow-up across the four groups. **D**: Comparison of EDSS at the last follow-up across the four groups after overlap weighting. ARR: Annualized Relapse Rate; EDSS: Expanded Disability Status Scale

We stratified the enrolled NMOSD patients into five groups on their management pathway as illustrated in section Method: NIS IV (*n* = 46), NNIS IV (*n* = 142), Transfer (*n* = 95), NIS LV (*n* = 141), and NNIS LV (*n* = 47). (Fig. 1) Analysis of the ARR at last follow revealed that patients in the NIS IV group demonstrated significantly lower rates (median: 0.39, IQR: 0.31–0.51, 95% CI: 0.32–0.45) compared to other groups: NNIS IV group (median: 0.53, IQR: 0.34–0.71, 95% CI: 0.47–0.57; NNIS LV group (median: 0.47, IQR: 0.32–0.86, 95% CI: 0.39–0.73; Transfer group (median: 0.53, IQR: 0.35–0.65, 95% CI: 0.47–0.58) (Fig. 2 A). Notably, patients in the Transfer group showed a marked improvement, with ARR significantly decreasing post-transfer (median: 0, IQR: 0-0.32, 95%CI: 0.0-0.15) compared to pre-transfer values (median: 1.71, IQR: 0.62-2.00, 95%CI: 1.33-2.00) (*p* < 0.001).

Evaluation of neurological disability using the EDSS demonstrated that NIS IV patients maintained significantly lower scores (median: 1.3, IQR: 1.0–2.0, 95% CI: 1.0–2.0) compared to other groups: NNIS IV group (median: 3.0, IQR: 2.0–5.0, 95% CI: 2.0-3.5; NNIS LV group (median: 3.0, IQR: 2.0–5.0, 95% CI: 2.0–4.0; Transfer group (median: 2.5, IQR: 2.0-3.5, 95% CI: 2.0–3.0). Furthermore, NIS LV patients exhibited lower EDSS scores (median: 2.0, IQR: 1.0-3.4, 95% CI: 2.0-2.3) compared to NNIS LV patients (median: 3.0, IQR: 2.0–5.0, 95% CI: 2.0–4.0). (Fig. 2 C).

Current clinical evidence suggests that monoclonal antibodies, including rituximab and inebilizumab, exhibit superior efficacy in reducing both ARR and neurological disability compared to conventional immunosuppressants in NMOSD patients. Our analysis revealed significantly higher administration rates of monoclonal antibodies in the NIS LV group compared to the NNIS LV group (38.29% vs. 12.77%, *p* = 0.002). Furthermore, NIS LV patients demonstrated a significantly higher rate of favorable prognosis, defined as an EDSS score of less than 5, at the final follow-up compared to NNIS LV patients (87.94% vs. 70.21%, *p* = 0.011).

After PSOW, all covariables were well balanced. Patients in NIS IV group still had the lowest ARR (median: 0.40, IQR: 0.31–0.53, 95% CI: 0.32–0.45) and EDSS (median: 2.0, IQR: 1.0–2.0, 95% CI: 1.0–2.0) at last follow-up among groups.(Fig. 2B and D).

### Factors associated with EDSS in NMOSD

**Fig. 3:**
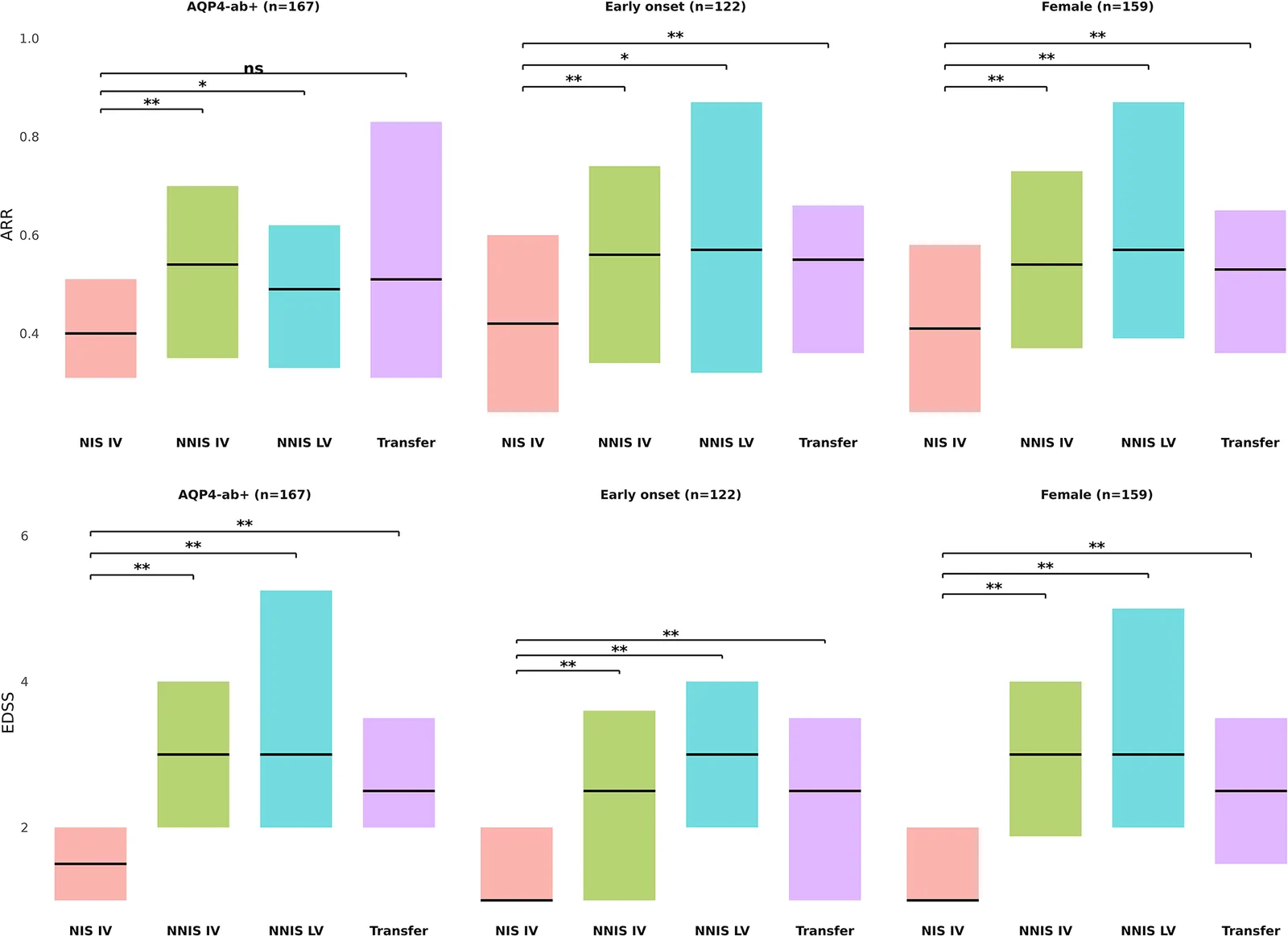
Sensitivity analyse. NIS IV: Initial visit in neuroimmunology subspecialty; NNIS IV: Initial visit in non-neuroimmunology subspecialty; NNIS LV: Last visit in non-neuroimmunology subspecialty; Transfer: a subset of NNIS IV patients later transferred their ongoing management to NIS. ARR: Annualized Relapse Rate; AQP4-ab：anti-aquaporin antibody; EDSS: Expanded Disability Status Scale

A ordinal logistic regression analysis was conducted to identify factors associated with EDSS at last follow. The univariable regression analysis revealed several significant factors: age of onset less than 50 years (*p* < 0.001), total number of attacks (*p* < 0.001), disease duration (*p* = 0.005), NIS IV (*p* = 0.016), NIS LV (*p* = 0.006), utilization of monoclonal antibody (*p* = 0.011) and disease duration (*p* = 0.010). Subsequent multivariable regression analysis indicated the earlier onset age (less than 50 years), patients in NIS IV and NIS LV had a lower risk to get the high EDSS, while more total attacks was associated with the high EDSS at last follow-up (Fig. 3).

### Sensitivity analysis

**Fig. 4:**
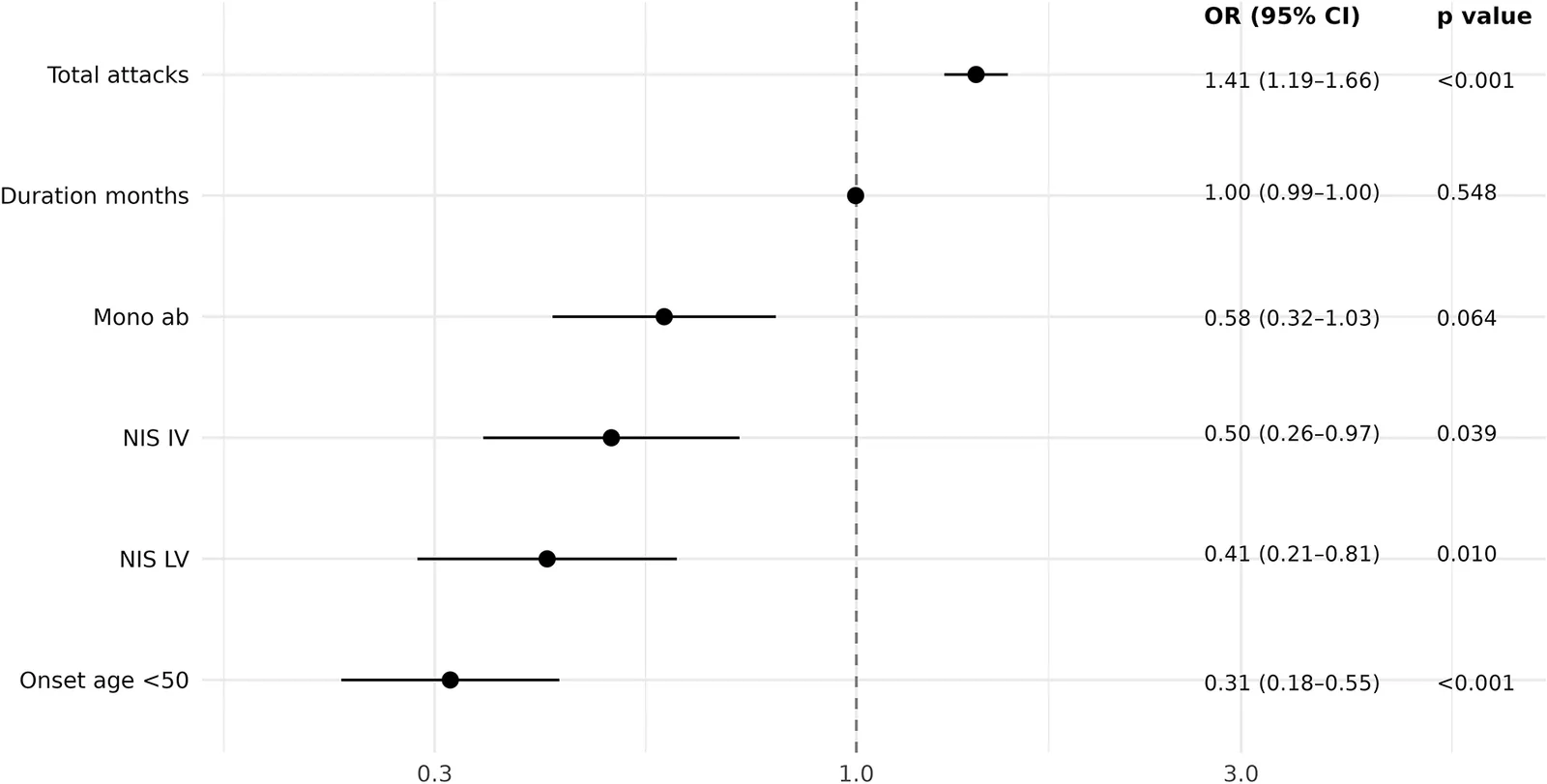
Factors affecting EDSS at last follow-up in patients with NMOSD. CI: Confidence interval; Mono ab: monoclonal antibody; NIS IV: Initial visit in neuroimmunology subspecialty; NIS LV: Last visit in neuroimmunology subspecialty; OR: Odds ratio

The results indicate that the sensitivity analyses for the three subsamples were consistent with the main analysis results. (Fig. 3; sTable. A) (Fig. 4).

## Discussion

Our findings demonstrated that patients with NMOSD who initially presented under NIS management exhibited lower ARR and EDSS, and results from PSOW further supported the robustness of our findings. Furthermore, patients who transfer from NNIS management to NIS showed significant reduction in ARR. Notably, patients who maintained consistent follow-up management within NIS demonstrated superior prognostic outcomes compared to those followed in NNIS.

NMOSD is a rare inflammatory demyelinating disease of the CNS, accounting for approximately one-third of CNS inflammation in Asian populations [13]. The importance of specialized management is further emphasized by our findings that patients managed by neuroimmunology subspecialists had significantly lower annualized relapse rates and higher utilization of monoclonal antibody therapies (39.72% vs. 12.77%, *p* = 0.001), which may correlated with better outcomes. The publication of the 2015 International Consensus Diagnostic Criteria for NMOSD marked a significant advancement in disease management, providing comprehensive guidance for diagnosis and treatment strategies [3]. Recent advances in understanding disease pathogenesis have led to the development and implementation of novel therapeutic agents, including rituximab, inebilizumab, and satralizumab [9–12, 14, 15]. These monoclonal antibodies have demonstrated superior efficacy compared to traditional immunosuppressants, particularly in reducing relapse frequency and mitigating neurological disability progression.

The increasing complexity of NMOSD management, particularly with the advent of targeted therapies and diagnosis, highlights the critical importance of subspecialty management. This is substantiated by our findings demonstrating that patients who transitioned from NNIS to NIS management exhibited marked clinical improvement, with significant reductions in post-transfer ARR (median: 1.71 vs. 0, *p* < 0.001). The superior outcomes observed in the NIS IV group, characterized by lower relapse rates and disability scores, demonstrate that specialized neuroimmunology management can effectively address the challenging relapsing course of NMOSD through optimized therapeutic selection.

A notable disparity exists in the implementation of advanced therapeutic strategies among non-neuroimmunology specialists, particularly regarding novel monoclonal antibody therapies. This management gap is especially problematic given that patients frequently present with diverse initial manifestations, including intractable hiccups, vomiting, pruritus, or vision loss, frequently leading to initial consultation with non-neurological specialists. The limited recognition of NMOSD among these practitioners can result in delayed diagnosis and treatment initiation. Such delays are particularly consequential given NMOSD’s characteristic presentation with cluster-like attacks in its early phases, potentially resulting in moderate to severe permanent neurological sequelae, with approximately 90% of cases following a relapsing disease course.

The complexity and heterogeneity of NMOSD manifestations [16], particularly in cases with atypical presentations and multiple autoimmune comorbidities, underscore the potential value of neuroimmunology subspecialty management in these patients. Neuroimmunology specialists are uniquely positioned to recognize and manage the diverse spectrum of clinical presentations, including rare manifestations [17, 18] such as brain cortical lesions, epileptic seizures, and intractable pruritus, which may be overlooked in routine clinical practice. Their expertise becomes particularly crucial in cases where NMOSD coexists with other autoimmune conditions, requiring careful balance of multiple immunological treatments. The comprehensive understanding of neuroimmunological mechanisms enables neuroimmunological specialists to make more informed decisions regarding diagnostic approaches and therapeutic strategies, potentially leading to earlier diagnosis, more targeted treatment interventions and management of drug related adverse events. Furthermore, their experience in managing complex autoimmune disorders may be instrumental in predicting and preventing potential complications, as well as optimizing long-term outcomes.

In accordance with the Neuromyelitis Optica Study Group (NEMOS) [14] study recommendations, long-term immunotherapy is mandatory for AQP4-ab-positive patients. In contrast, for double-negative NMOSD cases, long-term immunotherapy should be initiated following a relapse or severe initial attack. Regarding therapeutic transitions, monoclonal antibody therapy should be implemented upon failure of conventional immunosuppressive therapies, with the switching interval minimized to optimize outcomes. Our findings support the expeditious transition to monoclonal antibody therapy when conventional immunosuppressive treatments prove inadequate. This is evidenced by the higher rates of monoclonal antibody utilization observed among patients in the NIS IV (45.7%) and NIS LV (39.7%) groups, as well as those who transferred to NIS management (37.5%). Significantly, patients who transitioned from NNIS to NIS demonstrated enhanced clinical outcomes compared to those maintaining non-specialized management.

NIS training programs improve diagnostic accuracy, treatment optimization and patients outcome with neuroimmunological disease, including NMOSD. The development of neuroimmunology subspecialty training is crucial for advancing the field and improving patient management. By fostering expertise in this complex and evolving area, training programs can ensure that future specialists are well-equipped to address the challenges of neuroimmunological disorders and contribute to the broader healthcare system [19–22]. 

Several limitations should be considered when interpreting our findings. First, the retrospective design may introduce selection and recall biases. Our findings reflect practices at specific medical center, potentially limiting generalizability across diverse healthcare settings. Third, variations in patient follow-up duration may influence outcome assessments. Additionally, our study population may not fully represent the global NMOSD demographic spectrum, and geographic disparities in access to neuroimmunology subspecialists were not comprehensively addressed. Future research would benefit from large-scale, multicenter prospective studies to validate these findings across diverse healthcare environments.

## Conclusion

The study underscores the pivotal role of neuroimmunology subspecialty in NMOSD management, demonstrating that patients receiving management from neuroimmunology subspecialists, whether initially or through subsequent transfer, achieve better clinical outcomes, as evidenced by lower ARR and EDSS scores. These findings advocate for the increased acknowledgment of neuroimmunology subspecialists’ contributions to comprehensive NMOSD management.

## Supplementary Information

Below is the link to the electronic supplementary material.

Supplementary Material 1

## Data Availability

The datasets used and/or analyzed during the current study are available from the corresponding author upon reasonable request.
