# Supplementary material for: The role of neuroimmunology subspecialty in disease management of patients with neuromyelitis optica spectrum disorder
Source: Orphanet J Rare Dis. 2026 Jun 12;21:289. doi: 10.1186/s13023-026-04443-x (PMC13488285; doi:10.1186/s13023-026-04443-x)
Supplement: Supplementary file 1 [file 13023_2026_4443_MOESM1_ESM.docx]

|  | ARR | | | | | | | EDSS | | | | | | |
| --- | --- | --- | --- | --- | --- | --- | --- | --- | --- | --- | --- | --- | --- | --- |
|  | NIS IV | NNIS IV | NNIS LV | Transfer | p 1 | p2 | P3 | NIS IV | NNIS IV | NNIS LV | Transfer | p 1 | p 2 | P3 |
| Female (n=159) | 0.41±0.17 | 0.54 (0.37-0.73) | 0.57 (0.39-0.87) | 0.53 (0.36-0.65) | <0.001 | 0.003 | 0.003 | 1.0 (1.0-2.0) | 3.0 (1.9-4.0) | 3.0(2.0-5.0) | 2.5(1.5-3.5) | <0.001 | <0.001 | <0.001 |
| AQP4-ab seropositivy (n=167) | 0.40 (0.31-0.51) | 0.54 (0.35-0.70) | 0.49 (0.33-0.62) | 0.51 (0.31-0.83) | 0.003 | 0.026 | 0.069 | 1.5 (1.0-2.0) | 3.0 (2.0-4.0) | 3.0 (2.0-5.3) | 2.5 (2.0-3.5) | <0.001 | 0.004 | <0.001 |
| Early onset (n=122) | 0.42±0.18 | 0.56 (0.34-0.74) | 0.57 (0.32-0.87) | 0.55 (0.36-0.66) | 0.005 | 0.037 | 0.007 | 1.0 (1.0-2.0) | 2.5 (1.0-3.6) | 3.0(2.0-4.0) | 2.5(1.0-3.5 | <0.001 | <0.001 | <0.001 |

sTable. 1 Sensitivity analysis

AQP4-ab: Aquaporin4 antibody; ARR: Annualized Relapse Rate; EDSS: Expanded Disability Status Scale; NIS IV: Initial visit in neuroimmunology subspecialty; NIS LV: Last visit in neuroimmunology subspecialty; NNIS IV: Initial visit in non-neuroimmunology subspecialty; NNIS LV: Last visit in non-neuroimmunology subspecialty.

P1: comparison between NIS IV and NNIS IV; p2: comparison between NIS IV and NNIS LV; p3: comparison between NIS IV and Transfer;
